# Fast cycle of vagus nerve stimulation is associated with increased sleep‐related breathing disorder in patients with epilepsy

**DOI:** 10.1002/epi.70147

**Published:** 2026-02-13

**Authors:** Jacques‐François Massa, Nada El Youssef, Romain Carron, Pauline Garcia, Agnès Trebuchon, Bernard Giusiano, Fabrice Bartolomei, Angela Marchi, Isabelle Lambert

**Affiliations:** ^1^ Department of Epileptology and Brain Rhythmology Timone Hospital Marseille France; ^2^ Aix‐Marseille University INSERM, Institute of Systems Neuroscience Marseille France; ^3^ Medicosurgical Unit of Epilepsy, Functional and Stereotactic Neurosurgery, and Gamma Knife Radiosurgery La Timone University Hospital Marseille France

**Keywords:** AHI, drug‐resistant epilepsy, sleep apnea, VNS

## Abstract

**Objective:**

This work was undertaken to study the association between vagus nerve stimulation (VNS) parameters and the apnea–hypopnea index (AHI) measured by polysomnography in patients with drug‐resistant epilepsy.

**Methods:**

Patients with epilepsy who underwent polysomnography with an active VNS device between 2018 and 2023 were retrospectively included. VNS output current and cycle speed, defined as the interval between two stimulation‐ON (Stim‐ON) periods, were correlated with AHI. Demographic, clinical, and polysomnography characteristics were compared between patients with low (≤1.5 mA) or high (>1.5 mA) output current, and between patients with slow VNS cycle (interval between two Stim‐ON of >108 s) and fast cycle (interval between two Stim‐ON of ≤108 s). Multiadjusted linear regression analysis was performed.

**Results:**

Twenty‐two patients were included. Output current significantly correlated with AHI, but no difference was found between patients with low versus high output current. Patients with a fast VNS cycle had a significantly higher AHI than patients with a slow VNS cycle. After adjustment for age, number of antiseizure medications, body mass index, and gender, only the cycle speed remained significant, with no significant association with output current.

**Significance:**

Cycle speed, defined as a short interval between two stimulations, is associated with increased sleep‐related respiratory events in patients with epilepsy treated with VNS. Increasing the duration between two stimulations may help clinicians to reduce VNS‐induced sleep‐related breathing disorder.

**Key points**

- VNS may induce sleep‐related breathing disorder, with respiratory events occurring during the stimulation‐ON period.
- A shorter interval between two stimulation‐ON periods is associated with an increased apnea–hypopnea index.
- Increasing the interval between two stimulation‐ON periods may improve VNS‐induced sleep‐related breathing disorder.
- The effect of output current on upper airway and respiratory centers may be different according to acute or chronic stimulation.
- Prospective studies on VNS‐related changes of upper airway muscle contraction, ventilatory instability, and sleep instability are needed.

## 1 INTRODUCTION

Vagus nerve stimulation (VNS) is a neuromodulation therapy used in drug‐resistant epilepsy as an adjunctive therapy to antiseizure medication (ASM). The antiepileptic effect of VNS remains incompletely understood, but it may rely on a widespread cortical activation associated with a decrease of functional connectivity in the epileptogenic zone resulting from VNS‐related catecholamine release in the locus coeruleus.^1^, ^2^ VNS has been described to either disturb or improve sleep in patients with epilepsy with heterogeneous methods and results (see Seth et al.^3^ for review). The relationship between sleep and epilepsy is complex.^4^, ^5^, ^6^ As epileptic discharges and seizures may disrupt sleep,^7^ VNS may improve sleep because of decreased epilepsy‐induced sleep disruptions. Nevertheless, a specific action of VNS on the wake–sleep system has also been hypothesized, suggesting that VNS may modulate arousal during both sleep and wakefulness.^8^, ^9^, ^10^, ^11^, ^12^ Several studies have reported side effects of VNS therapy on breathing, with increased sleep‐related breathing disorder (SBD).^13^ VNS‐induced SBD is mostly represented by obstructive sleep apnea syndrome, but some other breathing patterns, such as central sleep apneas, stridor, or catathrenia, have also been reported.^14^, ^15^, ^16^ SBD is a condition associated with epilepsy worsening.^17^, ^18^, ^19^ Because sleep disorders are a frequent comorbidity and may worsen epilepsy, several recommendations and expert opinions have been proposed to help clinicians to search for and manage sleep comorbidities in patients with epilepsy.^20^, ^21^ For now, the mechanisms responsible for VNS‐induced SBD are poorly understood. VNS induces contraction of laryngeal muscles during stimulation‐ON (Stim‐ON), with vocal cord adduction that can be explored by awake or drug‐induced sleep laryngeal endoscopy.^14^, ^22^ However, VNS may also alter respiratory command, as central apneas during Stim‐ON have also been reported.^15^ Several retrospective studies have investigated the changes on polysomnographic sleep parameters before and after VNS treatment and found increased respiratory events.^13^, ^22^, ^23^, ^24^ Changes in different VNS parameters may improve the apnea–hypopnea index (AHI),^24^, ^25^ but these changes have mostly been described in case reports, some of which combine simultaneous changes in different parameters, making it harder to reach a conclusion.

In this retrospective monocentric study, we aimed to study the associations between two VNS parameters, output current and cycle speed, and the AHI, in patients treated with VNS therapy for drug‐resistant epilepsy.

## 2 MATERIALS AND METHODS

### 2.1 Patients

We retrospectively included all patients treated with VNS for drug‐resistant epilepsy who underwent polysomnography (PSG) between January 2018 and June 2023 at the Sleep Center, Epileptology Department, Timone Hospital, Marseille, France.

PSG was indicated during the epileptology clinical follow‐up to investigate complaints of sleep disturbances, excessive daytime sleepiness, or suspected nocturnal seizures.

Inclusion criteria were as follows: (1) patients with drug‐resistant epilepsy with an active VNS device, (2) age > 18 years, (3) full PSG with VNS stimulation artifact recorded either by chin electromyography (EMG) or neck electrodes.

Exclusion criteria were as follows: (1) artifacts preventing sleep or breathing analysis, (2) VNS stimulation signal not detected during sleep, (3) information about VNS cycle and output current not available, (4) PSG with continuous positive airway pressure (CPAP) or mandibular advancement device.

When several PSGs were performed for the same patient (after a change of VNS parameters), only the first PSG was considered for analysis.

Demographic data, type and number of ASMs, number of seizures during the past month, and questionnaires (Epworth Sleepiness Scale [ESS], Stop Bang, Insomnia Severity Index [ISI]) were collected.

This study was approved by the local ethics committee (PADS 24‐163dgd).

### 2.2 Sleep recordings

Video‐PSG was performed by Medatec or Embla SDx (Natus) devices with scalp electroencephalographic channels (F4, C4, O2, F3, C3, O1) in referential montage with contralateral mastoid electrodes (M1, M2), chin EMG, electro‐oculography, respiratory airflow, chest and abdomen inductance plethysmography, and anterior tibialis EMG electrodes.

Sleep scoring, microarousals, and sleep‐associated breathing and motor events were assessed according to American Academy of Sleep Medicine (AASM) recommendations^26^, ^27^ by board‐certified sleep specialists (N.E.Y., A.M., and I.L.).

As recommended by the French Sleep Society and the French Respiratory Society,^28^ AHI measured by PSG was the respiratory disturbance index defined as the total of apneas, hypopneas, and respiratory effort‐related arousals (RERAs) divided by the hours of total sleep time.

### 2.3 VNS parameters

**FIGURE 1:**
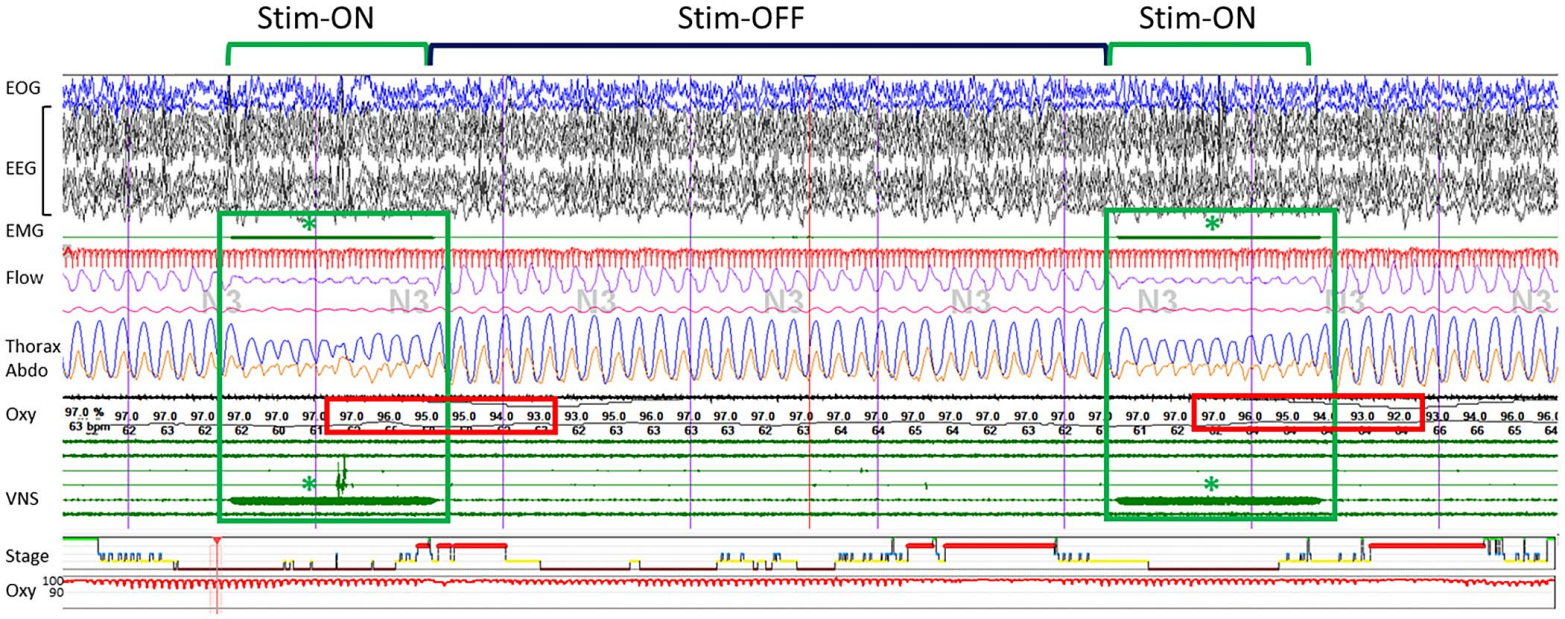
Example of a polysomnographic recording with vagus nerve stimulation (VNS). The stimulation‐ON (Stim‐ON) phase is represented by green boxes, and the stimulation artifact is marked with an asterisk. In this example, the Stim‐ON phase lasts 30 s, and the Stim‐OFF phase lasts 108 s. Note the decrease in flow amplitude and oxygen (Oxy) saturation during the Stim‐ON phase in the red boxes. Abdo, abdomen; EEG, electroencephalogram; EMG, electromyogram; EOG, electro‐oculogram.

VNS was provided with a VNS Therapy System (Demi‐pulse [model 103], Aspire SR [model 106], or Sentiva [model 1000], LivaNova). VNS parameters were defined according to the duration of the stimulation (Stim‐ON phase, in seconds), the interval between the offset of one Stim‐ON phase and the onset of the following Stim‐ON phase (Stim‐OFF phase, in seconds), the output current of stimulation during Stim‐ON phase (in milliamperes), and the frequency of the stimulation during the Stim‐ON phase (in hertz; Figure 1).

VNS cycle was defined as slow or fast, with a threshold defined according to the median duration of Stim‐OFF phase (108 s). VNS cycle was defined as slow if the Stim‐OFF phase was strictly greater than 108 s, and as fast if the Stim‐OFF phase was less than or equal to 108 s.

The duty cycle is a parameter that combines the duration of the Stim‐ON and Stim‐OFF phases. It indicates the stimulation cadence, which has been shown to be associated with VNS efficacy in treating epilepsy.^29^ Initial VNS parameters are typically set to a 10% duty cycle (30 s Stim‐ON and 5 min Stim‐OFF), which may later be increased by decreasing Stim‐OFF duration. Duty cycle was calculated as follows: (ON time + 2 × 2 s triangular ramps) / (ONTime + OFF time × 60) × 100%.^30^

The threshold between low and high output current was defined according to the median output current in our studied population (1.5 mA). Low output current was determined if VNS output current was less than or equal to 1.5 mA, and high output current if VNS output current was strictly greater than 1.5 mA.

SBD was considered to be VNS‐associated when more than 75% of respiratory events (apneas, hypopneas, or RERAS) occurred during the stimulation ON‐phase, not VNS‐associated when more than 75% of respiratory events were outside the Stim‐ON phase, and both VNS and not VNS‐associated if respiratory events occurred during Stim‐ON phase between 25% and 75% of cases. The choice of these thresholds is based on previous studies demonstrating the ineffectiveness of CPAP therapy for VNS‐associated SBD, which makes it necessary to distinguish between VNS‐associated and non‐VNS‐associated SBD.^31^, ^32^

### 2.4 Statistical analysis

Univariate analyses were used to compare patients and sleep characteristics according to VNS parameters, either the output current of VNS or the cycle speed. Considering the size of our population, we used nonparametric tests.

Nonparametric Mann–Whitney analysis was performed to compare AHI according to VNS parameters. The analyzed variables were age, gender, body mass index, VNS output current, VNS cycle, number of ASMs, VNS duration, microarousal index (total and respiratory), N3 sleep proportion, rapid eye movement (REM) sleep proportion, periodic limb movement index, and oxygen desaturation index ≥ 3%. Correlation between variables was performed by calculating the Spearman rank correlation coefficient. Multiadjusted analysis with a linear regression model was used to study the association between AHI and both patient and VNS characteristics. The variables considered for the multiadjusted analysis were cycle speed, output current, age, number of ASMs, body mass index, and gender.

Statistical analyses were performed with Jamovi (version 2.3).^33^

## 3 RESULTS

### 3.1 Patient characteristics

Thirty‐one PSGs were initially included. Nine PSGs were excluded (eight duplicates, one under CPAP therapy). Finally, 22 PSG were analyzed (10 women, 12 men; Table 1).

**TABLE 1:** Patient characteristics.

Characteristic	Value	Mean (SD)
Age, years	22	37.91 (12.9)
Gender, F/M	10/12	
Duration of epilepsy, years	19	22.58 (11.6)
Number of antiseizure medications	22	3.73 (1.1)
VNS duration, months	21	62.14 (42.6)
Number of monthly seizures	21	18.6 (22.9)
BMI	22	24.49 (4.8)
Stim‐OFF duration, s	22	127.36 (75.6)
Stim‐ON duration, s	22	27.9 (5.5)
ESS score	17	9.35 (4.4)
ISI score	14	9.64 (7.2)
Stop Bang score	17	2.35 (1.2)
AHI, /h	22	16.53 (14.6)
Microarousal index, /h	21	16.45 (14.3)
Respiratory microarousal index, /h	21	7.28 (6.6)
Periodic limb movement index, /h	22	2.57 (4.4)
N3 duration, min	22	62.73 (42)
REM duration, % of TST	22	13.05 (4.8)

Abbreviations: AHI, apnea–hypopnea index; BMI, body mass index; ESS, Epworth Sleepiness Scale; F, female; ISI, Insomnia Severity Index; M, male; N3, non‐REM sleep stage N3; REM, rapid eye movement sleep; Stim, stimulation; TST, total sleep time; VNS, vagus nerve stimulation.

All patients had left‐sided VNS. Given the small size of our population, the results are presented as the median (interquartile range [IQR]). Median age was 36.5 years (IQR = 28.3–46.8), with median body mass index of 24.1 (IQR = 21–27.1). Sixteen of 22 patients had focal epilepsy, three of 22 genetic generalized epilepsy, and three of 22 Lennox–Gastaut syndrome. The median number of ASMs was 3.5 (IQR = 3–4). Median VNS duration was 54 months (IQR = 39–81). Median VNS output current was 1.5 mA (IQR = 1.25–2), with VNS output current strictly >1.5 mA in 10 patients. Eight patients had slow cycle VNS (Stim‐OFF duration of 180 s for six patients, 300 s for two patients), and 14 patients had fast cycle VNS (Stim‐OFF duration of 108 s for seven patients, 66 s for three patients, 60 s for one patient, 48 s for one patient, 30 s for two patients). Median Stim‐OFF duration was 108 s (IQR = 66–180). The duration of stim‐ON phase was 30 s in 18 patients, 25 s in one patient, 21 s in one patient, 20 s in one patient, and 7 s in one patient. Stim‐OFF duration and VNS output current were not significantly correlated (Spearman, *ρ* = −.25, *p* = .260). Among the 19 patients with information on VNS frequency, 12 patients had VNS frequency of 20 Hz, two had 25 Hz, and five had 30 Hz. Pulse width was 250 μs in 20 patients (two had missing data). Median AHI was 14.2/h (IQR = 5–22, mean = 16.5, SD = 14.58). Among the 16 patients with AHI > 5/h, nine patients had VNS‐associated SBD, five patients did not have VNS‐associated SBD, and two patients had both VNS‐associated and non‐VNS‐associated SBD.

### 3.2 Univariate analysis

#### 3.2.1 VNS output current

**FIGURE 2:**
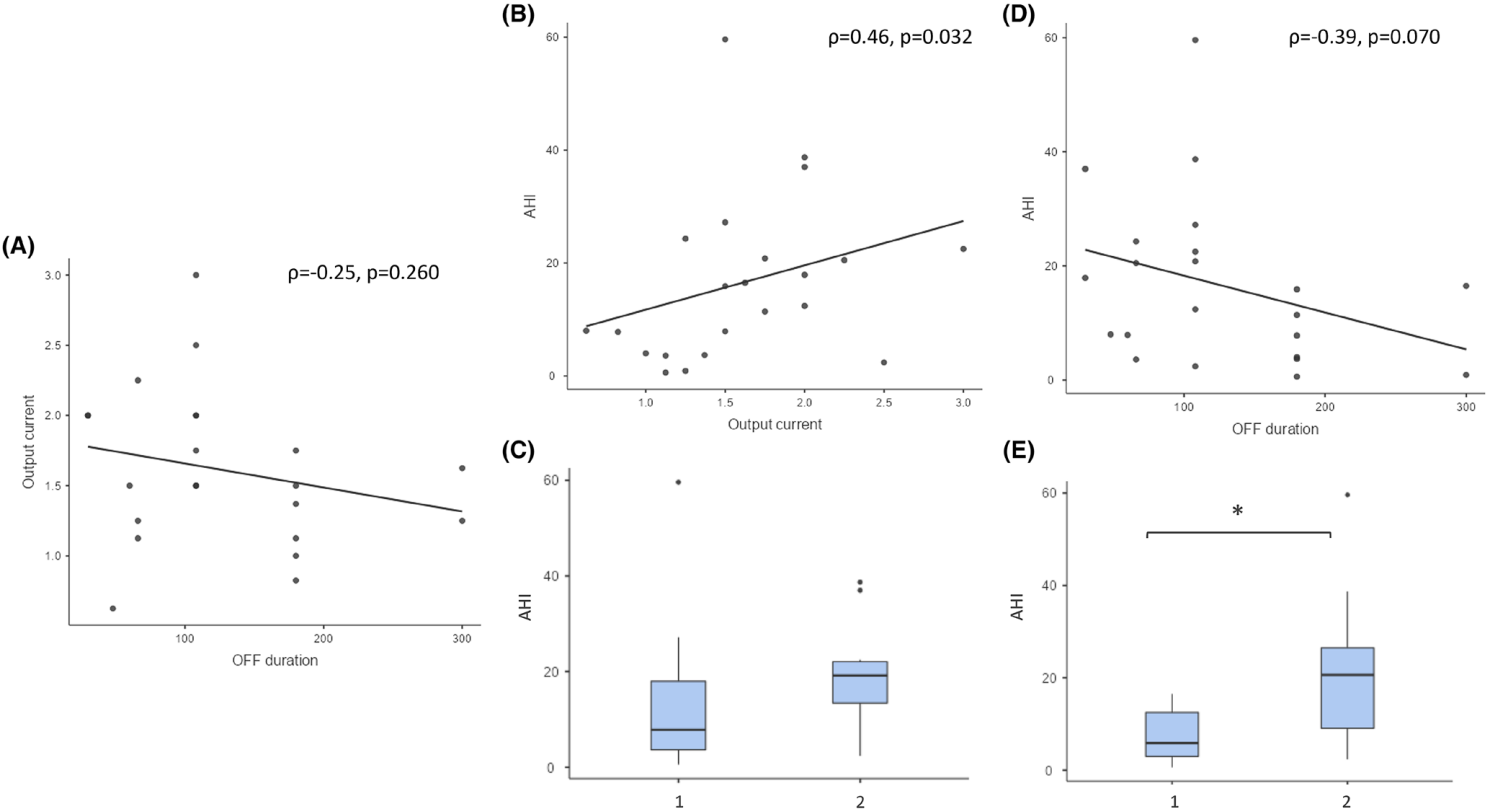
Variation of apnea–hypopnea index (AHI) according to vagus nerve stimulation (VNS) output current and cycle speed: univariate analysis. (A) No significant correlation between output current and stimulation‐OFF (Stim‐OFF) duration (Spearman, *ρ* = −.25, *p* = .260). (B, C) VNS output current. (B) AHI is positively correlated with output current (Spearman, *ρ* =.46, *p* = .032). (C) No significant difference of AHI according to low or high output current (>1.5 mA, group 2; ≤1.5 mA, group 1). (D, E) VNS cycle. (D) Negative correlation between AHI and Stim‐OFF duration, not reaching statistical significance (Spearman, *ρ* = −.39, *p* = .070). (E) AHI is significantly higher in patients with fast VNS cycle (≤108 s, group 2) compared to patients with slow VNS cycle (>108 s, group 1): mean = 21.6/h (SD = 15.64) versus 7.6/h (SD = 6.4), respectively, mean AHI difference of 14.3/h (Mann–Whitney, *p* = .013). Linear regression lines are only used for illustrative purposes.

There was no significant difference in AHI between patients with low and high VNS output current (Mann–Whitney, *p* = .107; Figure 2 and Table 2). We found no significant difference between patients with low or high VNS output current. However, we found a significant positive correlation between VNS output current and AHI (Spearman, *ρ* = .46, *p* = .032).

**TABLE 2:** Univariate comparisons according to VNS output current and cycle speed.

Characteristic	Output current					Cycle speed				
	*n*	Low	*n*	High	*p*	*n*	Slow	*n*	Fast	*p*
Age, years	12	36.25 (11.3)	10	39.9 (14.9)	.668	8	41.63 (10.4)	14	35.79 (14)	.152
BMI	12	23.42 (4.6)	10	25.78 (5)	.322	8	26.54 (5.6)	14	23.32 (4.1)	.172
Duration of epilepsy, years	10	21.4 (12.1)	10	23.8 (10.8)	.622	7	18.86 (7.4)	12	24.75 (13.2)	.309
VNS duration, months	11	53.2 (41.5)	10	72 (43.6)	.218	8	45.38 (42.2)	13	72.46 (41)	.076
Number of monthly seizures	11	20.8 (29.7)	10	16.1 (13.3)	.775	7	27.07 (35.4)	14	14.36 (13.2)	.734
Number of ASMs	12	3.92 (1)	10	3.5 (1.3)	.333	8	4 (1.3)	14	3.57 (1)	.41
ISI score	8	9.5 (8.1)	6	9.8 (6.6)	1	6	7.83 (8.5)	8	11 (6.3)	.649
ESS score	10	9.5 (5.6)	7	9.1 (2.3)	.805	7	10.29 (5.6)	10	8.7 (3.6)	.73
Stop Bang score	9	2.2 (1.1)	8	2.5 (1.3)	.763	4	1.75 (1)	13	2.54 (1.2)	.262
AHI, /h	12	13.6 (16.9)	10	20 (11.1)	.107	8	7.6 (6.4)	14	21.63 (15.6)	.013*
Microarousal index, /h	11	19.7 (17.3)	10	11.9 (10)	.341	7	11.03 (6.6)	14	19.16 (16.5)	.322
Respiratory microarousal index, /h	11	6.39 (6.6)	10	8.26 (6.9)	.597	7	3.6 (4.5)	14	9.12 (6.9)	.048*
Periodic limb movement index, /h	12	2.1 (3.2)	10	3.2 (5.6)	.782	8	3.83 (6.3)	14	1.85 (2.8)	.971
N3 duration, min	12	62.8 (44.4)	10	62.7 (41.4)	.692	8	37.81 (32.4)	14	76.96 (41)	.031*
REM duration, % of TST	12	13.1 (4.3)	10	13 (5.6)	.947	8	14.25 (3.1)	14	12.37 (5.5)	.495
ODI 3%, /h	12	7.9 (12.1)	10	7.5 (9.8)	.508	8	5.5 (8.3)	14	9 (12.2)	.373

*Note*: Values are presented as mean (SD).

Abbreviations: AHI, apnea–hypopnea index; ASM, antiseizure medication; BMI, body mass index; ESS, Epworth Sleepiness Scale; ISI, Insomnia Severity Index; N3, non‐REM sleep stage N3; ODI 3%, oxygen desaturation index ≥ 3%; REM, rapid eye movement sleep; TST, total sleep time; VNS, vagus nerve stimulation.

* *p* < .05.

Considering PSG parameters, no significant difference was found between low and high output current regarding sleep structure (N3 and REM sleep durations) and microarousal index.

#### 3.2.2 VNS cycle

AHI was significantly higher in patients with fast VNS cycle compared to patients with slow VNS cycle (mean = 21.6/h [SD = 15.64] versus 7.6/h [SD = 6.4] respectively, with mean AHI difference of 14.3/h [Mann–Whitney, *p* = .013]). In line with this result, respiratory microarousal index was significantly higher in patients with fast VNS cycle compared to slow VNS cycle (*p* = .048). We also found a negative correlation between Stim‐OFF phase duration and AHI, which did not reach statistical significance (Spearman, *ρ* = −.39, *p* = .070). No correlation was found between duty cycle and AHI (Spearman, *ρ* = .29, *p* = .188).

There was no significant difference in age, epilepsy duration, number of monthly seizure frequency, number of ASMs, ISI, ESS, or Stop Bang scores. Although not reaching statistical significance, there was an increased VNS duration in patients with fast cycles (*p* = .076). Considering PSG parameters, no significant difference was found between slow and fast cycles regarding REM sleep proportion and total microarousal index. We found an increase of N3 sleep in patients with fast VNS cycle (mean = 76.96 min, SD = 41) compared to patients with slow VNS cycle (mean = 37.81 min, SD = 32.4).

### 3.3 Multiple regression analysis

Linear multiple regression analyses adjusted for age, gender, body mass index, VNS output current, VNS cycle speed, and number of ASMs showed significant associations between AHI and age (effect estimation = .674, SD = .272, *p* = .027), and between AHI and VNS cycle, with a significantly higher AHI in patients with fast VNS cycle compared to patients with VNS slow cycle (effect estimation = 18.609, SD = 6.272, *p* = .005; Table 3). Due to its discontinuous distribution, we considered cycle speed to be a categorical variable. The linear multiple regression analysis with VNS cycle speed as a continuous variable is presented in the Supporting Information. To prevent a threshold effect and considering the significant correlation between VNS output current values and AHI, we considered continuous values of VNS output current instead of output current‐based groups for this analysis. No significant association was found with VNS output current.

**TABLE 3:** Multiple linear regression analysis.

Predictor	Effect estimation	Standard error	*t*	*p*
Age, years	.674	.272	2.479	.026*
Number of ASMs	3.237	2.920	1.109	.285
BMI	.464	.633	.734	.474
Cycle speed				
Fast vs. slow	18.609	6.251	2.977	.009*
Gender				
Male vs. female	−5.672	5.495	−1.032	.318
Output current	2.125	4.943	.430	.673

*Note*: Linear regression analysis: significant predictors of AHI in the studied population are age (effect estimation = .67, *p* = .026) and speed of cycle (effect estimation = 18.6, *p* = .009). No significant association was found between AHI and vagus nerve stimulation output current (continuous values, mA) after adjustment. Abbreviations: AHI, apnea–hypopnea index; ASM, antiseizure medication; BMI, body mass index.

* *p* < .05.

## 4 DISCUSSION

In our retrospective study, we investigated the association between VNS parameters and obstructive sleep apnea syndrome evaluated with PSG in a group of 22 patients with drug‐resistant epilepsy. Our findings revealed that a fast VNS cycle was significantly associated with a higher AHI. This association is particularly noteworthy, as sleep‐related breathing disturbances emerging after VNS initiation may compromise both seizure control and quality of life. These insights could guide clinicians in optimizing VNS settings to balance therapeutic benefits with sleep safety.

### 4.1 Fast VNS cycle is associated with higher AHI

**FIGURE 3:**
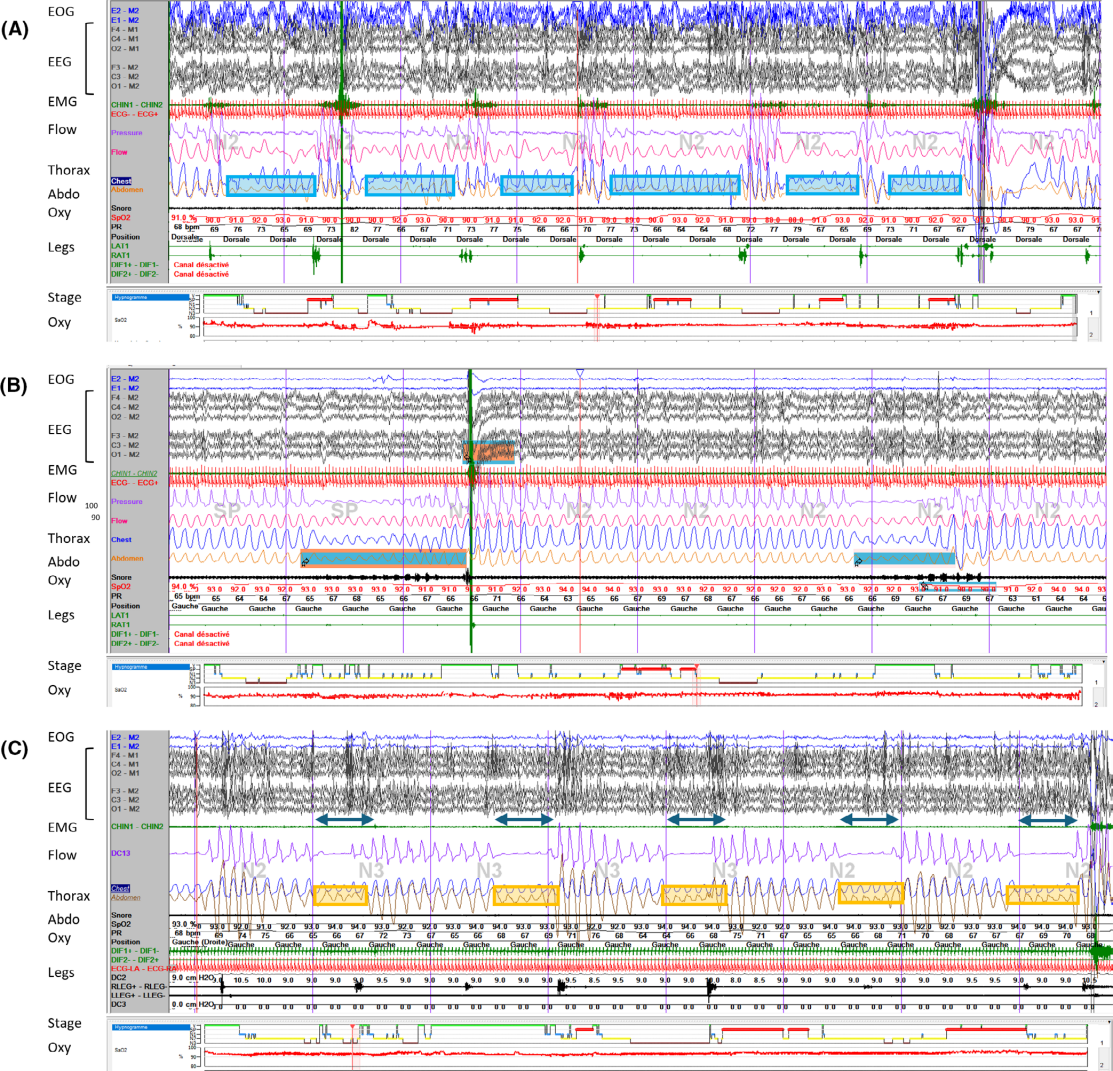
Case report of a patient with vagus nerve stimulation (VNS)‐associated and non‐VNS‐associated sleep‐related breathing disorder, showing increased apnea–hypopnea index (AHI) despite continuous positive airway pressure (CPAP) therapy after VNS cycle speed was increased (4‐min polysomnography illustration). (A) First polysomnography before VNS implantation: positional obstructive sleep apnea with an AHI of 25.6/h (hypopnea index = 22.4/h, apnea = 3.2/h, AHI in the supine position = 38.5/h, AHI not in the supine position: 6.6/h). (B) Second polysomnography with VNS (output current = 1.5 mA, frequency = 15 Hz, pulse width = 130 μs, stimulation‐ON [Stim‐ON] duration = 30 s, Stim‐OFF duration = 108 s): severe obstructive sleep apnea with an AHI of 38.2/h (hypopnea index = 33.4/h, apnea = 4.8/h), without positional component (AHI in the supine position = 42.6/h, AHI not in the supine position = 29.8/h). (C) Third polysomnography under 10 cm H_2_O CPAP therapy with VNS (output current = .75 mA, frequency = 20 Hz, pulse width = 250 μs, Stim‐ON duration = 14 s, Stim‐OFF duration = 30 s): severe obstructive sleep apnea with an AHI of 57.6/h (hypopnea index = 5.8/h, apnea index = 51.9/h, respiratory arousals = 7.9/h), without positional component (AHI in the supine position = 68/h, AHI not in the supine position = 51/h). Abdo, abdomen; EEG, electroencephalogram; EMG, electromyogram; EOG, electro‐oculogram; Oxy, oxygen.

In our population, we found that a fast VNS cycle was associated with a clinically significant increase in AHI. This result suggests that decreasing the time between two Stim‐ON phases may facilitate the occurrence of significant respiratory events included in AHI. We hypothesize that closely spaced VNS stimulations may disrupt ventilatory stability by increasing loop gain, thereby promoting the occurrence of significant respiratory events during sleep. The loop gain is a concept inspired by electrical engineering. This is one of the putative mechanisms involved in obstructive sleep apnea (OSA) (see^34^ for review). It involves a reflex loop between peripheral chemoreceptors and central respiratory centers (controller gain), considering the influence of other factors influencing ventilatory response, such as pCO2 value, cardiac ejection fraction, or sleep stages, with higher loop gain being associated with increased significant respiratory events. VNS has a direct action on vocal cord adduction,^22^ but it may also have an indirect effect on brainstem respiratory centers. Approximately 80% of vagus nerve afferent fibers terminate in the nucleus of tractus solitarius, which belongs to the dorsal respiratory group responsible for inspiration control in the brainstem.^2^, ^35^, ^36^ Therefore, a fast VNS cycle may induce ventilatory instability and increased loop gain by increasing controller gain. As it is estimated that approximately 30% of SBD cases are due to high loop gain, this hypothesis may explain why anatomical factors such as obesity are not sufficient to anticipate the risk of VNS‐induced SBD. A better defining of anatomical and nonanatomical factors associated with SBD in candidates for VNS therapy may help quantify the risk of VNS‐induced SBD. As VNS has been associated with increased arousal,^8^ we may hypothesize that ventilatory instability is caused by increased arousals during sleep. However, our results do not support this hypothesis, because most of the respiratory events of our patients are not associated with arousals. Furthermore, in our population, patients with fast VNS cycles have increased AHI, but they also have longer N3 sleep duration, which is associated with more stable sleep. The relationship between SBDs and arousal threshold is complex and not fully understood.^37^, ^38^, ^39^, ^40^ It is possible that sedative ASMs raised the arousal threshold in our patients; however, this parameter was not specifically assessed. Overall, our findings suggest that the increased AHI observed with fast VNS cycling is more likely driven by ventilatory instability rather than sleep instability, as illustrated in a case report (Figure 3).

### 4.2 High VNS output current and AHI risk in our population

Although univariate analysis revealed a positive correlation between VNS output current and AHI, multivariate analysis did not show a significant difference according to output current values. This contrasts with previous studies and case reports suggesting that reducing VNS output current may alleviate symptoms of sleep‐disordered breathing.^14^, ^23^, ^24^, ^41^ However, in some of the previously published case studies, several VNS parameters could be simultaneously changed with output current, such as stimulation frequency^24^ or Stim‐OFF duration.^14^ Decreasing VNS frequency may be associated with reducing respiratory events,^25^, ^42^ with no improvement after output current decrease in some reports from the same series. VNS output current may be monitored with laryngeal muscle evoked potentials (LMEPs), which have been shown to be associated with VNS efficacy in epilepsy.^43^ VNS output current appears to be efficient at low output current; however, some data suggest that its effect on nerve fibers may decrease after 1 year of VNS therapy compared to acute VNS therapy.^44^ In our study, all our patients had chronic VNS therapy, with a mean VNS duration of 62 months. Therefore, we cannot exclude that the effect of output current on laryngeal muscle, which is one of the putative mechanisms of VNS‐induced SBD, has already decreased with chronic stimulation, although it may be significant in acute stimulation. However, high interindividual variability has also been found in LMEPs of chronically stimulated patients, when considering recruitment curves of LMEP amplitude according to increasing levels of output current.^45^

### 4.3 Hypothetical mechanisms of VNS‐induced respiratory events

Based on our results and studies published by other teams, we hypothesize that VNS‐related respiratory events may result from different mechanisms, possibly in combination. Output current may be particularly relevant to laryngeal motility and may cause respiratory complaints, such as laryngeal constriction and stridor, during both wakefulness and sleep. High VNS cycle speed may increase ventilatory instability by impairing ventilatory command, also known as controller gain. Finally, VNS may lower the arousal threshold, inducing sleep fragmentation and increasing AHI through hypopnea‐related arousals. However, the arousal‐inducing effect of VNS may be counterbalanced by the higher arousal thresholds induced by some ASMs.

### 4.4 Are all VNS‐related respiratory events clinically significant?

In our study, patients were referred for PSG because of clinical symptoms suggestive of SBD or uncontrolled epilepsy. The increase in respiratory events in the fast VNS group was not associated with increased clinical scores assessing insomnia (ISI) or daytime sleepiness (ESS) in our population. Furthermore, the respiratory arousal index and oxygen desaturation index were quite low compared to the total count of respiratory events, which were mostly represented by apneas not associated with arousal or oxygen desaturation. These results may question the clinical relevance of systematically searching for VNS‐associated respiratory events in patients with epilepsy in clinical practice. In AHI, only respiratory events defined according to AASM classification are included, with well‐documented clinical consequences.^46^, ^47^ The VNS Stim‐ON phase is often associated with changes in breathing patterns during sleep that do not meet the criteria for respiratory events validated in AHI, as described by Garrett et al.,^48^ with VNS settings close to those used in our population. They proposed to include these breathing patterns in the definition of sleep‐related respiratory events. For now, there is no evidence that these subtle changes in breathing patterns may have clinical consequences. Furthermore, the mechanisms inducing epilepsy worsening by SBD are not yet well understood. Besides negative impact on cardiovascular health,^49^ hypoxemia has been suggested to increase neuronal and epileptic excitability in a rodent model of epilepsy exposed to prolonged hypoxemia.^50^ In our population, SBD was not associated with a high rate of oxygen desaturations, and so far, no data have supported that considering respiratory events with oxygen desaturation < 3%, as suggested by Nobili et al.,^48^ may be clinically relevant.

### 4.5 Limitations and strengths

Our study has several limitations. The monocentric retrospective nature of the study has induced a possible bias of recruitment and has limited an exhaustive collection of VNS parameters. Furthermore, the studied population size is quite small, with quite young and not obese patients, which may limit the generalization of the results. At last, the transversal nature of the study does not provide information about the efficacy of parameter changes. However, to the best of our knowledge, this is the first study that systematically studied the association between VNS parameters and sleep‐related respiratory events. These results may provide guidance for adapting VNS parameters in patients with VNS‐related SBD, although future prospective studies are needed to better phenotype baseline breathing pattern during sleep and investigate the role of each VNS parameter in acute and chronic stimulation.

## 5 CONCLUSIONS

In summary, our study demonstrates that a fast VNS cycle—characterized by shorter OFF periods between stimulations—is associated with a significant increase in sleep‐related respiratory events, independent of stimulation output current. These findings help to refine our understanding of VNS‐induced SBD and highlight a specific parameter—cycle speed—as a modifiable contributor to respiratory side effects.

## AUTHOR CONTRIBUTIONS

Jacques‐François Massa collected and analyzed data and wrote the manuscript. Nada El Youssef analyzed data and revised the manuscript. Pauline Garcia and Agnès Trebuchon revised the manuscript. Romain Carron and Fabrice Bartolomei contributed to the design of the study, analysis of the data, and interpretation of the results, and revised the manuscript. Bernard Giusiano analyzed the data and revised the manuscript. Angela Marchi conceptualized and designed the study, analyzed the data, and revised the manuscript. Isabelle Lambert conceptualized and designed the study, analyzed the data, and wrote the manuscript.

## CONFLICT OF INTEREST STATEMENT

R.C. has received honoraria from LivaNova for sharing expertise on VNS surgical techniques during symposia. F.B. has received honoraria from LivaNova for giving talks at conferences and for participation on a board of experts. The remaining authors have no conflicts of interest. We confirm that we have read the Journal's position on issues involved in ethical publication and affirm that this report is consistent with those guidelines.

## Supporting information


Table S1.

## Data Availability

The data that support the findings of this study are available from the corresponding author, I.L., upon reasonable request.

## References

[1] Collins L , Boddington L , Steffan PJ , McCormick D . Vagus nerve stimulation induces widespread cortical and behavioral activation. Curr Biol. 2021;31(10):2088–2098.e3. 33740425 10.1016/j.cub.2021.02.049

[2] Carron R , Roncon P , Lagarde S , Dibué M , Zanello M , Bartolomei F . Latest views on the mechanisms of action of surgically implanted cervical vagal nerve stimulation in epilepsy. Neuromodulation. 2023;26(3):498–506. 36064522 10.1016/j.neurom.2022.08.447

[3] Seth J , Couper RG , Burneo JG , Suller Marti A . Effects of vagus nerve stimulation on the quality of sleep and sleep apnea in patients with drug‐resistant epilepsy: a systematic review. Epilepsia. 2024;65(1):73–83. 37899679 10.1111/epi.17811

[4] Nobili L , Frauscher B , Eriksson S , Gibbs SA , Halasz P , Lambert I , et al. Sleep and epilepsy: a snapshot of knowledge and future research lines. J Sleep Res. 2022;31(4):e13622. 35487880 10.1111/jsr.13622 PMC9540671

[5] Malow BA . The interaction between sleep and epilepsy. Epilepsia. 2007;48(Suppl 9):36–38. 18047600 10.1111/j.1528-1167.2007.01400.x

[6] El Youssef N , Marchi A , Bartolomei F , Bonini F , Lambert I . Sleep and epilepsy: a clinical and pathophysiological overview. Rev Neurol (Paris). 2023;179(7):687–702. 37598088 10.1016/j.neurol.2023.07.006

[7] Peter‐Derex L , Klimes P , Latreille V , Bouhadoun S , Dubeau F , Frauscher B . Sleep disruption in epilepsy: ictal and interictal epileptic activity matter. Ann Neurol. 2020;88(5):907–920. 32833279 10.1002/ana.25884

[8] Rizzo P , Beelke M , De Carli F , Canovaro P , Nobili L , Robert A , et al. Chronic vagus nerve stimulation improves alertness and reduces rapid eye movement sleep in patients affected by refractory epilepsy. Sleep. 2003;26(5):607–611. 12938816 10.1093/sleep/26.5.607

[9] Winter Y , Sandner K , Bassetti CLA , Glaser M , Ciolac D , Ziebart A , et al. Vagus nerve stimulation for the treatment of narcolepsy. Brain Stimul. 2024;17(1):83–88. 38184192 10.1016/j.brs.2024.01.002

[10] Rembado I , Song W , Su DK , Levari A , Shupe LE , Perlmutter S , et al. Cortical responses to vagus nerve stimulation are modulated by brain state in nonhuman primates. Cereb Cortex. 2021;31(12):5289–5307. 34151377 10.1093/cercor/bhab158 PMC8567998

[11] Ravan M , Begnaud J . Investigating the effect of short term responsive VNS therapy on sleep quality using automatic sleep staging. IEEE Trans Biomed Eng. 2019;66(12):3301–3309. 30869604 10.1109/TBME.2019.2903987

[12] Hallböök T , Lundgren J , Köhler S , Blennow G , Strömblad LG , Rosén I . Beneficial effects on sleep of vagus nerve stimulation in children with therapy resistant epilepsy. Eur J Paediatr Neurol. 2005;9(6):399–407. 16257548 10.1016/j.ejpn.2005.08.004

[13] Kim JS , Lee DE , Bae H , Song JY , Yang KI , Hong SB . Effects of Vagus nerve stimulation on sleep‐disordered breathing, daytime sleepiness, and sleep quality in patients with drug‐resistant epilepsy. J Clin Neurol. 2022;18(3):315–322. 35589319 10.3988/jcn.2022.18.3.315 PMC9163944

[14] Oliveira Santos M , Bentes C , Teodoro T , Moreira S , Marques M , Tomé D , et al. Complex sleep‐disordered breathing after vagus nerve stimulation: broadening the spectrum of adverse events of special interest. Epileptic Disord. 2020;22(6):790–796. 33337335 10.1684/epd.2020.1223

[15] Forde IC , Mansukhani MP , Kolla BP , Kotagal S . A potential novel mechanism for vagus nerve stimulator‐related central sleep apnea. Children. 2017;4(10):86. 28961186 10.3390/children4100086 PMC5664016

[16] St Louis EK , Faber K . Reversible sleep‐related stridor during vagus nerve stimulation. Epileptic Disord. 2010;12(1):76–80. 20189905 10.1684/epd.2010.0294 PMC2958706

[17] Malow BA , Levy K , Maturen K , Bowes R . Obstructive sleep apnea is common in medically refractory epilepsy patients. Neurology. 2000;55(7):1002–1007. 11061259 10.1212/wnl.55.7.1002

[18] Foldvary‐Schaefer N , Andrews ND , Pornsriniyom D , Moul DE , Sun Z , Bena J . Sleep apnea and epilepsy: who's at risk? Epilepsy Behav. 2012;25(3):363–367. 23103311 10.1016/j.yebeh.2012.08.032

[19] Foldvary‐Schaefer N , Grigg‐Damberger M . Sleep and epilepsy: what we know, don't know, and need to know. J Clin Neurophysiol. 2006;23(1):4–20. 16514348 10.1097/01.wnp.0000206877.90232.cb

[20] Nobili L , de Weerd A , Rubboli G , Beniczky S , Derry C , Eriksson S , et al. Standard procedures for the diagnostic pathway of sleep‐related epilepsies and comorbid sleep disorders: a European academy of neurology, European Sleep Research Society and international league against epilepsy‐Europe consensus review. J Sleep Res. 2020;29(6):e13184. 32959468 10.1111/jsr.13184

[21] Nobili L , Beniczky S , Eriksson SH , Romigi A , Ryvlin P , Toledo M , et al. Expert opinion: managing sleep disturbances in people with epilepsy. Epilepsy Behav. 2021;124:108341. 34619543 10.1016/j.yebeh.2021.108341

[22] Zambrelli E , Saibene AM , Furia F , Chiesa V , Vignoli A , Pipolo C , et al. Laryngeal motility alteration: a missing link between sleep apnea and vagus nerve stimulation for epilepsy. Epilepsia. 2016;57(1):e24–e27. 26589721 10.1111/epi.13252

[23] Voges BR . Bi‐level VNS therapy with different therapy modes at night and daytime improves seizures and quality of life in a patient with drug‐resistant epilepsy. Epilepsy Behav Rep. 2023;24:100633. 38045989 10.1016/j.ebr.2023.100633 PMC10692657

[24] Salvadé A , Ryvlin P , Rossetti AO . Impact of vagus nerve stimulation on sleep‐related breathing disorders in adults with epilepsy. Epilepsy Behav Rep. 2018;79:126–129. 10.1016/j.yebeh.2017.10.040 29287215

[25] Dye TJ , Hantragool S , Carosella C , Huang G , Hossain MM , Simakajornboon N . Sleep disordered breathing in children receiving vagus nerve stimulation therapy. Sleep Med. 2021;79:101–106. 33485258 10.1016/j.sleep.2020.12.021

[26] Berry RB , Budhiraja R , Gottlieb DJ , Gozal D , Iber C , Kapur VK , et al. Rules for scoring respiratory events in sleep: update of the 2007 AASM manual for the scoring of sleep and associated events. Deliberations of the sleep apnea definitions task force of the American Academy of sleep medicine. J Clin Sleep Med. 2012;8(5):597–619. 23066376 10.5664/jcsm.2172 PMC3459210

[27] Berry RB , Brooks R , Gamaldo C , Harding SM , Lloyd RM , Quan SF , et al. AASM scoring manual updates for 2017 (version 2.4). J Clin Sleep Med. 2017;13(5):665–666. 28416048 10.5664/jcsm.6576 PMC5406946

[28] Société de Pneumologie de Langue Française , Société Française d'Anesthésie Réanimation , Société Française de Cardiologie , Société Française de Médecine du Travail , Société Française d'ORL, Société de Physiologie , et al. Recommendations for clinical practice. Obstructive sleep apnea hypopnea syndrome in adults. Rev Mal Respir. 2010;27(7):806–833. 20863987 10.1016/j.rmr.2010.05.011

[29] Heck C , Helmers SL , DeGiorgio CM . Vagus nerve stimulation therapy, epilepsy, and device parameters: scientific basis and recommendations for use. Neurology. 2002;59(6_Suppl_4):S31–S37. https://www.neurology.org/doi/10.1212/WNL.59.6_suppl_4.S31 12270966 10.1212/wnl.59.6_suppl_4.s31

[30] Fahoum F , Boffini M , Kann L , Faini S , Gordon C , Tzadok M , et al. VNS parameters for clinical response in epilepsy. Brain Stimul. 2022;15(3):814–821. 35643390 10.1016/j.brs.2022.05.016

[31] Ebben MR , Sethi NK , Conte M , Pollak CP , Labar D . Vagus nerve stimulation, sleep apnea, and CPAP titration. J Clin Sleep Med. 2008;4(5):471–473. 18853706 PMC2576315

[32] Oh DM , Johnson J , Shah B , Bhat S , Nuoman R , Ming X . Treatment of vagus nerve stimulator‐induced sleep‐disordered breathing: a case series. Epilepsy Behav Rep. 2019;12:100325. 31497754 10.1016/j.ebr.2019.100325 PMC6719281

[33] About – jamovi. https://www.jamovi.org/about.html

[34] Antonaglia C , Citton GM , Soave S , Salton F , Ruaro B , Confalonieri P , et al. Deciphering loop gain complexity: a primer for understanding a pathophysiological trait of obstructive sleep apnea patients. Respir Med. 2024;234:107820. 39332779 10.1016/j.rmed.2024.107820

[35] González‐García M , Carrillo‐Franco L , Morales‐Luque C , Dawid‐Milner MS , López‐González MV . Central Autonomic mechanisms involved in the control of laryngeal activity and vocalization. Biology. 2024;13(2):118. 38392336 10.3390/biology13020118 PMC10886357

[36] Henry TR . Therapeutic mechanisms of vagus nerve stimulation. Neurology. 2002;59(6 Suppl 4):S3–S14. 10.1212/wnl.59.6_suppl_4.s3 12270962

[37] Younes M . Role of arousals in the pathogenesis of obstructive sleep apnea. Am J Respir Crit Care Med. 2004;169(5):623–633. 14684560 10.1164/rccm.200307-1023OC

[38] Younes M , Ostrowski M , Atkar R , Laprairie J , Siemens A , Hanly P . Mechanisms of breathing instability in patients with obstructive sleep apnea. J Appl Physiol. 2007;103(6):1929–1941. 17823298 10.1152/japplphysiol.00561.2007

[39] Sands SA , Terrill PI , Edwards BA , Taranto Montemurro L , Azarbarzin A , Marques M , et al. Quantifying the arousal threshold using polysomnography in obstructive sleep apnea. Sleep. 2018;41(1):zsx183. 29228393 10.1093/sleep/zsx183 PMC5804982

[40] Antonaglia C , Passuti G . Obstructive sleep apnea syndrome in non‐obese patients. Sleep Breath. 2022;26(2):513–518. 34324126 10.1007/s11325-021-02412-1 PMC9130173

[41] Sponaugle A , Stainman RS , Carosella CM . Reduced VNS settings paradoxically decreases seizure burden in a patient following resolution of sleep disordered breathing. Epilepsy Behav Rep. 2025;31:100778. 40475325 10.1016/j.ebr.2025.100778 PMC12139479

[42] Malow BA , Edwards J , Marzec M , Sagher O , Fromes G . Effects of vagus nerve stimulation on respiration during sleep: a pilot study. Neurology. 2000;55(10):1450–1454. 11094096 10.1212/wnl.55.10.1450

[43] Berger A , Cerra M , Joris V , Danthine V , Macq B , Dricot L , et al. Identifying responders to vagus nerve stimulation based on microstructural features of thalamocortical tracts in drug‐resistant epilepsy. Neurotherapeutics. 2024;21(5):e00422. 38964949 10.1016/j.neurot.2024.e00422 PMC11579871

[44] Bouckaert C , Raedt R , Larsen LE , El Tahry R , Gadeyne S , Carrette E , et al. Laryngeal muscle‐evoked potential recording as an indicator of vagal nerve fiber activation. Neuromodulation Technol Neural Interface. 2022;25(3):461–470. 10.1016/j.neurom.2022.01.014 35177376

[45] Vespa S , Stumpp L , Bouckaert C , Delbeke J , Smets H , Cury J , et al. Vagus nerve stimulation‐induced laryngeal motor evoked potentials: a possible biomarker of effective nerve activation. Front Neurosci. 2019;13:880. 31507360 10.3389/fnins.2019.00880 PMC6718640

[46] Haba‐Rubio J , Marti‐Soler H , Tobback N , Andries D , Marques‐Vidal P , Waeber G , et al. Sleep characteristics and cognitive impairment in the general population: the HypnoLaus study. Neurology. 2017;88(5):463–469. 28039311 10.1212/WNL.0000000000003557

[47] Gervès‐Pinquié C , Bailly S , Goupil F , Pigeanne T , Launois S , Leclair‐Visonneau L , et al. Positive airway pressure adherence, mortality, and cardiovascular events in patients with sleep apnea. Am J Respir Crit Care Med. 2022;206(11):1393–1404. 35816570 10.1164/rccm.202202-0366OC

[48] Garrett AL , Burch J , Zhang Y , Li H , Sundar KM , Farney RJ . Depicting and defining sleep disturbed breathing associated with vagal nerve stimulation. Sleep Med. 2023;110:68–75. 37542741 10.1016/j.sleep.2023.07.034

[49] Azarbarzin A , Sands SA , Stone KL , Taranto‐Montemurro L , Messineo L , Terrill PI , et al. The hypoxic burden of sleep apnoea predicts cardiovascular disease‐related mortality: the osteoporotic fractures in men study and the sleep heart health study. Eur Heart J. 2019;40(14):1149–1157. 30376054 10.1093/eurheartj/ehy624 PMC6451769

[50] Villasana‐Salazar B , Hernández‐Soto R , Guerrero‐Gómez ME , Ordaz B , Manrique‐Maldonado G , Salgado‐Puga K , et al. Chronic intermittent hypoxia transiently increases hippocampal network activity in the gamma frequency band and 4‐aminopyridine‐induced hyperexcitability in vitro. Epilepsy Res. 2020;166:106375. 32745888 10.1016/j.eplepsyres.2020.106375

