# Supplementary material for: Fast cycle of vagus nerve stimulation is associated with increased sleep‐related breathing disorder in patients with epilepsy
Source: Epilepsia. 2026 Feb 13;67(5):2347–57. doi: 10.1002/epi.70147 (PMC13179660; doi:10.1002/epi.70147)
Supplement: Supplementary file 1 — Table S1. [file EPI-67-2347-s001.docx]

|  | | | | | | | | | |
| --- | --- | --- | --- | --- | --- | --- | --- | --- | --- |
| **Predictor** | | **Effect estimation** | | **Standard Error** | | **t** | | **p** | |
| Age |  | 0.8457 |  | 0.2962 |  | 2.855 |  | **0.012** | ***** |
| Gender |  | -6.6069 |  | 5.5226 |  | -1.196 |  | 0.250 |  |
| BMI |  | 0.0885 |  | 0.6314 |  | 0.140 |  | 0.890 |  |
| Number of ASM |  | 1.5557 |  | 2.8537 |  | 0.545 |  | 0.594 |  |
| Output current |  | 4.4982 |  | 4.7742 |  | 0.942 |  | 0.361 |  |
| OFF duration |  | -0.1136 |  | 0.0401 |  | -2.835 |  | **0.013** | ***** |
|  | | | | | | | | | |

Supplementary Table 1

Linear regression analysis with VNS speed cycle considered in continuous variables

*p<0.05
